# Supplementary material for: Quantum absorption refrigerator with trapped ions
Source: Nat Commun. 2019 Jan 14;10:202. doi: 10.1038/s41467-018-08090-0 (PMC6331551; doi:10.1038/s41467-018-08090-0)
Supplement: Supplementary file 1 — Supplementary Information [file 41467_2018_8090_MOESM1_ESM.pdf]

Supplementary Information - Quantum Absorption Refrigerator with Trapped Ions. Maslennikov et al.

Supplementary Note 1. Trilinear Hamiltonian.

A detailed derivation of the coupling between the modes of motion in the trapped ion system is given in Supplementary Reference 1. Here we show how the trilinear Hamiltonian (Eq. (1) in the main text) arises from the Coulomb interaction between the three ions in our experiment.

The potential energy of the ions in the trap is

$$V = \frac{1}{2}\sum_{i=1}^{3} m(\omega_x^2 x_i^2 + \omega_y^2 y_i^2 + \omega_z^2 z_i^2) + \sum_{\substack{i,j=1 \\ i>j}}^{3} \frac{e^2}{4\pi\epsilon_0} \frac{1}{\sqrt{(x_i - x_j)^2 + (y_i - y_j)^2 + (z_i - z_j)^2}} \tag{1}$$

where (x_i, y_i, z_i) denote the coordinates of the ion i, $\omega_x, \omega_y, \omega_z$ are trap frequencies for a single ion, e is the electron charge, and ϵ_0 is the dielectric vacuum permittivity. In a cigar-shaped trapping potential, where $\sqrt{12/5}\omega_z < \omega_x, \omega_y$, the ions align along the z axis. The equilibrium position of the ions is then determined by the equations $\partial V/\partial z_i = 0$ at $x_i = y_i = 0$, which results in the distance $z_0 = \left(5e^2/16\epsilon_0 m\omega_z\right)^{1/3}$ between neighboring ions.

For small displacements from equilibrium $\chi_i = \{x_1, x_2, x_3, y_1, y_2, y_3, z_1 + z_0, z_2, z_3 - z_0\}$, we can expand the potential energy as $V^{(h)} = \sum_{i,j} \frac{1}{2} \frac{\partial^2 V}{\partial \chi_i \partial \chi_j} \chi_i \chi_j$. The corresponding harmonic equations of motion for the displacements are

$$m\frac{d^2\chi_i}{dt^2} - \sum_j \frac{1}{2} \frac{\partial^2 V}{\partial \chi_i \partial \chi_j} \chi_j = 0. \tag{2}$$

Solutions have the form $\chi_j = A_j \exp(i\omega_j t)$, where the mode frequencies ω_k are determined by the

Mode frequency	Eigenmode	Normal mode coordinate	Name
Axial modes			
ω_z	$\frac{1}{\sqrt{3}}(1,1,1)$	$\zeta_1 = \frac{1}{\sqrt{3}}(z_1+z_2+z_3)$	centre of mass
$\sqrt{3}\omega_z$	$\frac{1}{\sqrt{2}}(1,0,-1)$	$\zeta_2 = \frac{1}{\sqrt{2}}(z_1-z_3+2z_0)$	rocking
$\sqrt{29/5}\omega_z$	$\frac{1}{\sqrt{6}}(1,-2,1)$	$\zeta_3 = \frac{1}{\sqrt{6}}(z_1-2z_2+z_3)$	zig-zag (hot)
Radial modes, y direction			
ω_y	$\frac{1}{\sqrt{3}}(1,1,1)$	$\zeta_4 = \frac{1}{\sqrt{3}}(y_1+y_2+y_3)$	centre of mass
$\sqrt{\omega_y^2-\omega_z^2}$	$\frac{1}{\sqrt{2}}(1,0,-1)$	$\zeta_5 = \frac{1}{\sqrt{2}}(y_1-y_3)$	rocking
$\sqrt{\omega_y^2-12\omega_z^2/5}$	$\frac{1}{\sqrt{6}}(1,-2,1)$	$\zeta_6 = \frac{1}{\sqrt{6}}(y_1-2y_2+y_3)$	zig-zag
Radial modes, x direction			
ω_x	$\frac{1}{\sqrt{3}}(1,1,1)$	$\zeta_7 = \frac{1}{\sqrt{3}}(x_1+x_2+x_3)$	centre of mass
$\sqrt{\omega_x^2-\omega_z^2}$	$\frac{1}{\sqrt{2}}(1,0,-1)$	$\zeta_8 = \frac{1}{\sqrt{2}}(x_1-x_3)$	rocking (work)
$\sqrt{\omega_x^2-12\omega_z^2/5}$	$\frac{1}{\sqrt{6}}(1,-2,1)$	$\zeta_9 = \frac{1}{\sqrt{6}}(x_1-2x_2+x_3)$	zig-zag (cold)

Supplementary Table 1: Motional modes of three ions.

characteristic equation

$$\det\left[-m\omega_i^2\delta_{ij} + \sum_j \frac{1}{2}\frac{\partial^2 V}{\partial\chi_i\partial\chi_j}\right] = 0. \tag{3}$$

For the linear ion crystal, the motional components of the ions along the principal axes of the trap are decoupled from each other. The resulting mode frequencies and eigenmodes of the oscillations are listed in Supplementary Table 1.

Higher order anharmonic terms in the Taylor expansion of the potential energy can couple these normal modes of motion. However, since the energy of the Coulomb interaction depends only on the relative position of the ions, the centre of mass modes are not coupled to any other modes. On the other hand, the "rocking" and "zigzag" modes for axial and radial directions can be coupled.

To calculate the coupling between the three motional modes of interest (marked as hot, work, and cold in Supplementary Table 1), we introduce the normal mode coordinates $\zeta_i = \sum_k e_k^{(i)} \chi_k$, with $e^{(i)}$ the eigenvector for the normal mode i. The anharmonic terms of lowest order in the Taylor expansion of the potential energy with respect to these coordinates are then $V^{(3)} = \sum_{ijk} \frac{1}{6} \frac{\partial^3 V}{\partial\zeta_i \partial\zeta_j \partial\zeta_k} \zeta_i \zeta_j \zeta_k$. For the quantum model, we replace the coordinates by $\hat{\zeta}_i = \sqrt{\hbar/2m\omega_i}(\hat{a}_i^\dagger + \hat{a}_i)$, with $\hat{a}_i$ ($\hat{a}_i^\dagger$) the phonon annihilation (creation) operator for mode i, m the ion mass, and $\hbar$ the Plank constant. Finally, when a resonance condition of the form $\omega_i = \omega_j + \omega_k$ is met, we may use the rotating wave approximation and keep only resonant terms in the interaction Hamiltonian; they describe the exchange of energy between the modes.

For the cold, hot, and work mode we then obtain Eq. (1) of the main text, $\hat{H} = \hbar\xi(\hat{a}_\mathrm{h}^\dagger \hat{a}_\mathrm{c} \hat{a}_\mathrm{w} + \hat{a}_\mathrm{h} \hat{a}_\mathrm{c}^\dagger \hat{a}_\mathrm{w}^\dagger)$, with the coupling constant

$$\xi = \left(\frac{\partial^3 V}{\partial\zeta_3 \partial\zeta_8 \partial\zeta_9}\right) \sqrt{\frac{\hbar}{8m^3 \omega_\mathrm{h} \omega_\mathrm{w} \omega_\mathrm{c}}} = \frac{9\omega_z^2}{5z_0} \sqrt{\frac{\hbar}{m\omega_\mathrm{h} \omega_\mathrm{w} \omega_\mathrm{c}}}. \tag{4}$$

Coulomb interaction can also induce nonlinear coupling between pairs of modes of the form $(\hat{a}_i \hat{a}_j^{\dagger 2} + \hat{a}_i^\dagger \hat{a}_j^2)$. In particular, the axial "rocking" mode can be coupled to either "rocking" or "zigzag" radial modes. However, the mode frequencies are chosen such that the required resonance conditions ($\omega_i = 2\omega_j$) is not satisfied and therefore such couplings do not result in observable effects. In Supplementary Table 2 we give the mode parameters that were used in the experiment, and in Supplementary Figure 1 we show the frequency spectrum of the radial modes.

Mode name	Mode frequency $\omega/(2\pi)$ (MHz)[1]		Vacuum Rabi frequency, $\Omega/(2\pi)$ (kHz)	Decay rate, $\gamma/(2\pi)$ (Hz)	Lamb-Dicke parameter
**Figures 2 and 3 (a-g) (high mode frequency set)**					
Axial modes					
centre of mass	0.561		6.67(6)		0.171
rocking	0.987		7.1(7)		0.130
zig-zag (hot)	1.371		4.155(5)	3.9(8)	0.110
Radial modes, y direction					
	off-resonant	resonant			off-resonant
centre of mass	0.912	0.938	3.9(2)		0.135
rocking	0.710	0.745	6.2(4)		0.153
zig-zag	0.233	0.316	2.7(3)		0.268
Radial modes, x direction					
	off-resonant	resonant			off-resonant
centre of mass	1.044	1.025	4.3(2)		0.128
rocking (work)	0.880	0.852	5.064(3)	27(5)	0.140
zig-zag (cold)	0.564	0.521	3.197(1)	20(2)	0.179
**Figures 3 (h-l) and 4 (low mode frequency set)**					
Axial modes					
centre of mass	0.418		8.5(1.1)		0.198
rocking	0.732		7.63(6)		0.15
zig-zag (hot)	1.024		3.797(5)	33(10)	0.128
Radial modes, y direction					
	off-resonant	resonant			off-resonant
centre of mass	0.683	0.702	3.11(20)		0.156
rocking	0.535	0.558	6.36(17)		0.177
zig-zag	0.195	0.242	2.90(2)		0.292
Radial modes, x direction					
	off-resonant	resonant			off-resonant
centre of mass	0.773	0.765	3.823(1)		0.147
rocking (work)	0.651	0.636	5.105(5)	15(8)	0.160
zig-zag (cold)	0.414	0.389	3.204(3)	13(6)	0.200

[1] The off-resonant mode frequencies are obtained from blue sideband scans after Sisyphus + sideband cooling. In this case, the values include the AC Stark shift between $|\downarrow\rangle$ and $|\uparrow\rangle$ states. Frequencies of "hot", "work" and "cold" modes are obtained by coherent excitation of motion from state $|a\rangle \equiv |S_{1/2}, F = 1, m_F = +1\rangle$ with the running optical lattice. Since the resonant mode frequencies cannot be measured directly, we provide the calculated values, using single ion frequencies.

Supplementary Table 2: Experimental parameters of the motional modes.

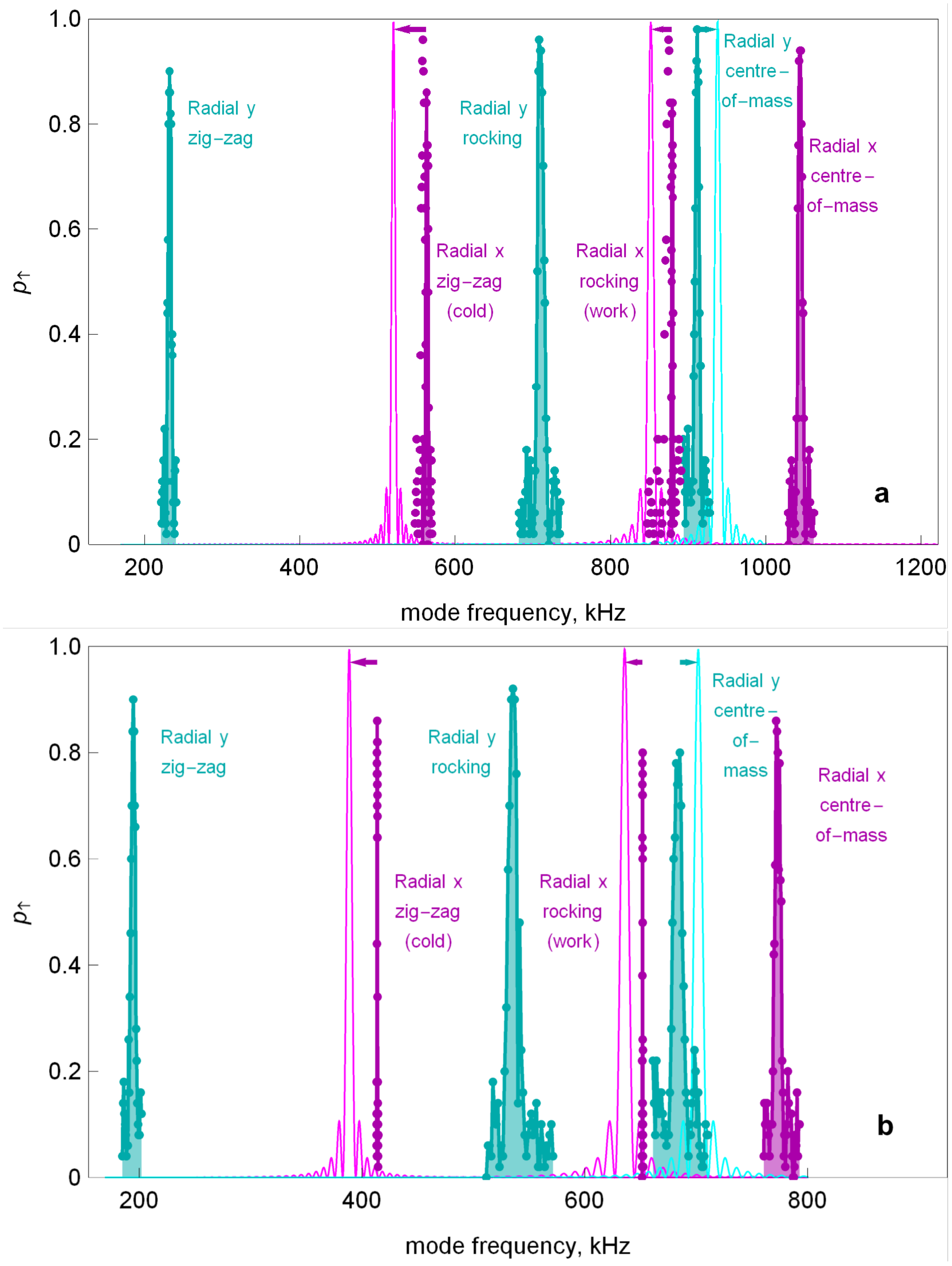

Supplementary Figure 1: Radial motional mode spectra after Sisyphus and sideband cooling. Experimental data points are measured as described in the supplementary text. The magenta (cyan) plots correspond to oscillation modes along x (y) trap axis. The arrows show the controlled displacement of the modes to the frequency values for which the resonance condition $\omega_{\mathrm{h}} = \omega_{\mathrm{w}} + \omega_{\mathrm{c}}$ is satisfied (solid lines, calculated from single ion frequencies). The data is presented for both high **(a)** and low **(b)** values of single ion trap frequencies (see Methods).

Supplementary Note 2. Measurement of mode frequencies.

We measure the mode frequencies utilizing two different methods: If high precision is not required, the mode frequencies are determined by frequency scan of blue sideband taken after Sisyphus and sideband cooling. However, the measured frequencies are affected by the fourth order Stark shift [2].

To avoid such effects for the modes used as refrigerator bodies, we start with the ions in the ground state of motion and optically pump bright ion to the internal state $|0\rangle$. Then we transfer it to state $|a\rangle$, apply a periodic dipole force using a running optical lattice, transfer it back to state $|0\rangle$ and measure the population of the mode by applying a red sideband pulse followed by detection of ion internal state. The frequency of the running optical lattice at which the mode is resonantly excited determines the mode frequency.

Supplementary Note 3. Calibration of the state preparation

The reproducible operation of the refrigerator requires careful calibration of the initial mean phonon numbers of the motional mode. Below we describe the procedures for preparation of thermal and squeezed thermal states and methods employed for the calibration.

Reconstruction of the phonon number distribution for the calibration procedure To reconstruct the phonon number distribution, we drive the blue sideband transition and measure the tem-

poral evolution of internal state of the ion. The data is then fitted to a function of the form [3]

$$p_{\uparrow}(t) = \frac{a}{2}\left(1 - \sum_{n=0} p(n)\cos\left(\Omega_{n,n+1}\, t\right) e^{-\gamma_n\, t}\right) + b, \tag{5}$$

where $p(n)$ is the expected population distribution for the target quantum state of the mode, $\Omega_{n,n+1} = \sqrt{n+1}\,\Omega_{0,1}$ is the state dependent Rabi frequency and $\gamma_n = \sqrt{n+1}\,\gamma_0$ is the decoherence rate [3]. Parameters a, b are introduced to account for the imperfect phonon detection efficiency and state detection background. The results of the fit are used to calibrate the preparation procedure for all the states used in the experiment.

Thermal state During the preparation of a thermal state, each step (see Methods) displaces the ions motional state by length α in a random direction in phase space. After N steps, the expected displacement from the origin is $\sqrt{N}\,\alpha$, which corresponds to $\bar{n} = N|\alpha|^2$ phonons, where $|\alpha|^2 = \bar{m}$ is the mean phonon number of coherent state after one step [4]. Adding the initial phonon population $\bar{n}_0$ after the imperfect sideband cooling [5] then gives

$$\bar{n} = \bar{n}_0 + N\bar{m}. \tag{6}$$

To experimentally verify this equation, we extract $\bar{m}$ and $\bar{n}$ by fitting the temporal evolution of coherent states and thermal states to Supplementary Equation 5, where the expected population distributions are

$$p(n) = \frac{\bar{m}^n\, e^{-\bar{m}}}{n!}, \tag{7}$$

and

$$p(n) = \frac{\bar{n}^n}{(\bar{n}+1)^{n+1}}, \tag{8}$$

respectively. The results are shown in Supplementary Figure 2, where the hot mode excitation is taken as an example. They are consistent with the prediction of Supplementary Equation 6.

Squeezing operation The squeezing operation on the work mode can be independently calibrated using squeezed vacuum state, which is prepared by starting from the vacuum state and then applying the optical dipole force at twice the trap frequency for some time. The expected population distribution of the squeezed vacuum state is restricted to even number states

$$p(2n) = \frac{(2n)!\,\mathrm{sech}(r)\tanh^{2n} r}{(2^n\, n!)^2}. \tag{9}$$

The squeezing parameter r is linearly proportional to the duration of the applied force [3], as shown in Supplementary Figure 3**a**.

To demonstrate the coherence of the squeezing operation, we apply a second squeezing pulse with the same pulse amplitude as the first one but $180°$ out of phase. We verify that this pulse brings the ions back to ground state with probability of more than 80% for $r = 1.2$.

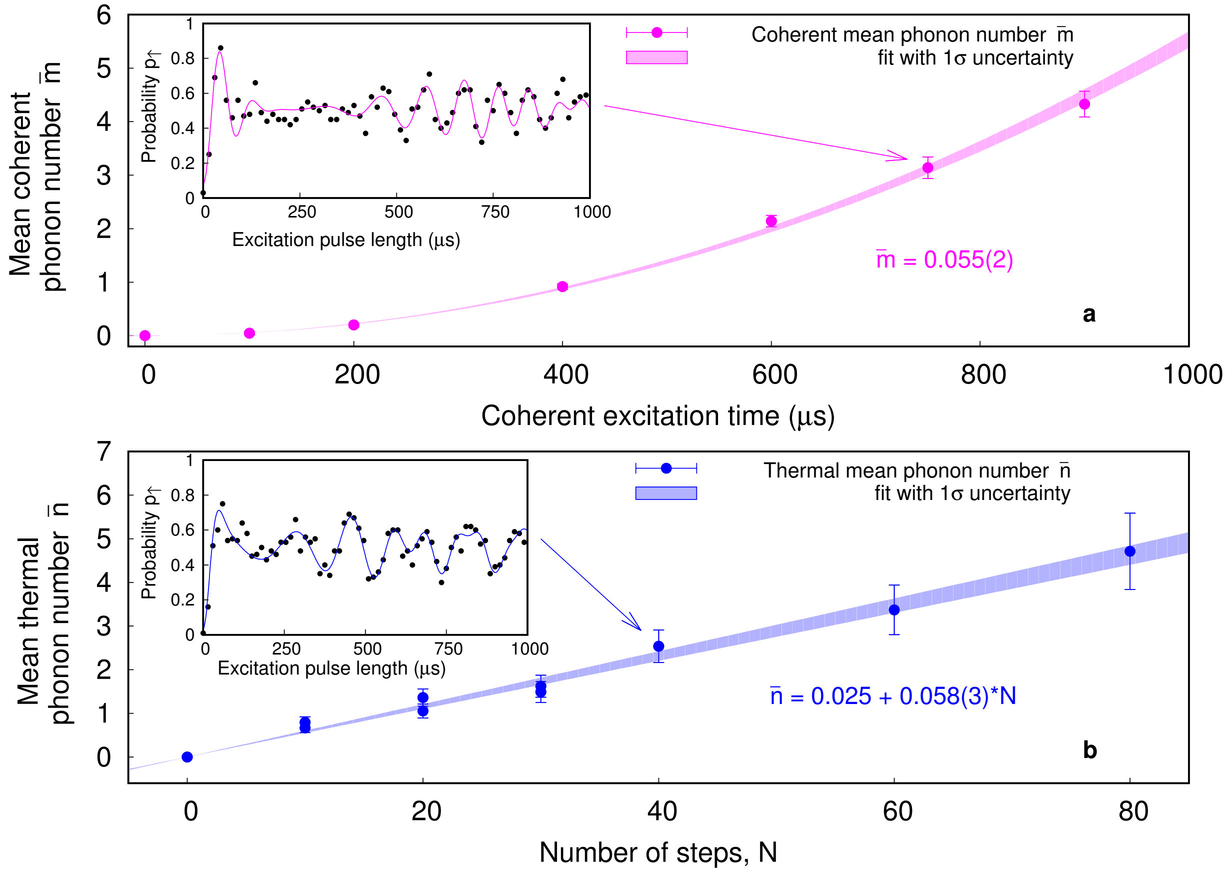

Supplementary Figure 2: **Thermal state preparation. a.** Mean phonon number of the coherent states as a function of excitation time. It is fitted to the function of the form $\bar{m}_t = \beta \times t^2$ to extract $\bar{m} = \beta \times (100\,\mu s)^2$ in Supplementary Equation 6. **b.** Mean phonon number dependence on the number of preparation steps. The linear fit yields the mean phonon number of the thermal state according to Supplementary Equation 6. The insets show the internal state evolution versus blue sideband excitation pulse length for coherent (**a**) and thermal (**b**) states. The fits to Supplementary Equation 5 with Poissonian (Supplementary Equation 7) and thermal (Supplemenary Equation 8) distributions yield the mean phonon numbers and their errors.

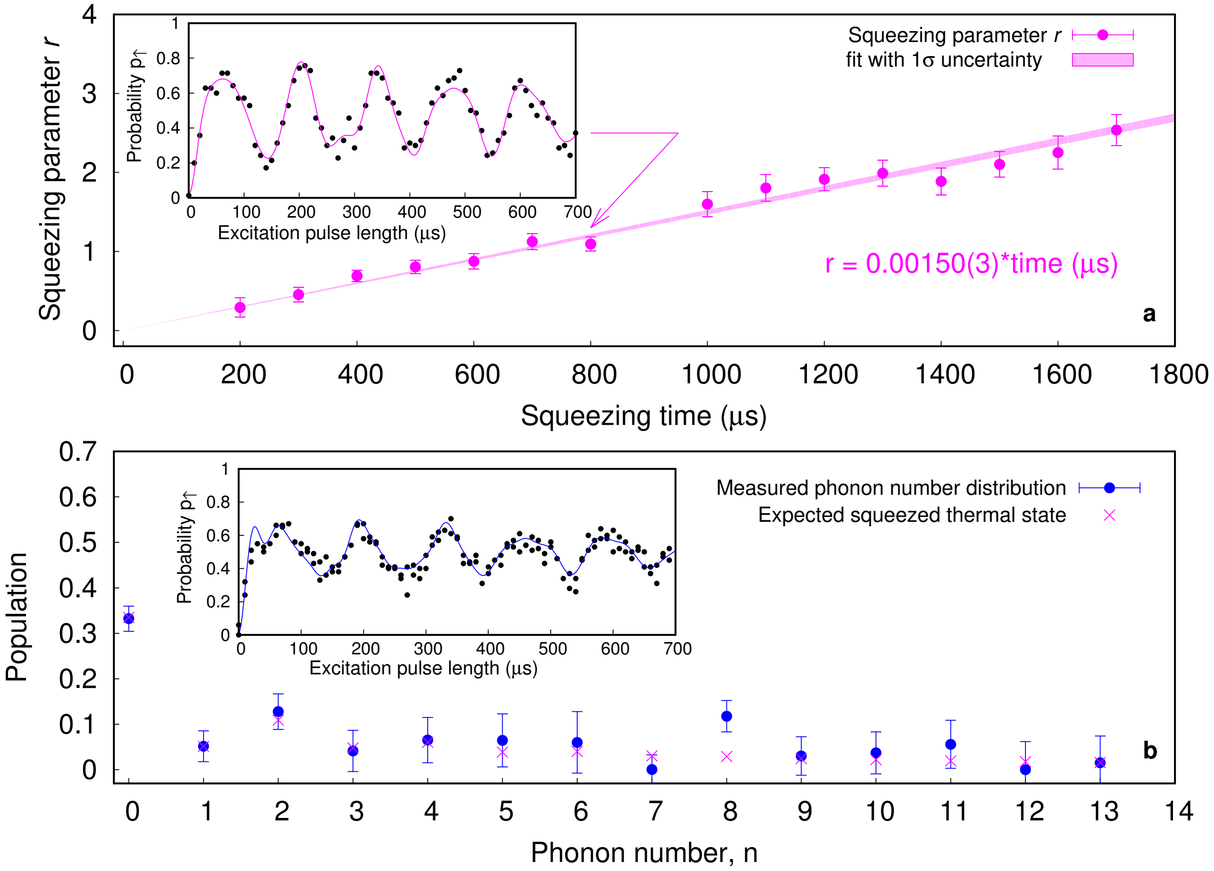

Supplementary Figure 3: **Squeezed vacuum and squeezed thermal state preparation. a.** Squeezing parameter r in the work mode as a function of squeezing operation time t applied to the vacuum state. The magenta dots are the experimental data and the magenta line is a fit using $r = \rho \times t$. The inset shows the internal state evolution as a function of the blue sideband pulse duration, measured for a squeezing time of $800\,\mu s$. The magenta curve is the fit with Supplementary Equation 9 which yields squeezing parameter $r = 1.09(9)$. Again, the vertical error bars are given by the errors of the fit (see inset). **b.** The phonon number population distribution of the squeezed thermal state. The distribution reconstructed (blue dots) from the fit of the experimental data (inset) where all the $p(n)$ are allowed to vary independently is compared to the distribution calculated from the expected values for $r = 1.2$ and $\bar{n} = 0.77$ (magenta crosses). The error bars represent the standard error of the fit used to obtain $p(n)$ values.

Squeezed thermal state The population distribution of squeezed thermal state with squeezing parameter r and initial phonon number $\bar{n}$ is [6,7]

$$p(n) = \sum_{m=0} \frac{\bar{n}^m}{(\bar{n}+1)^{m+1}} D_n^{SN}(m,r), \tag{10}$$

where

$$D_n^{SN}(m,r) = \begin{cases} \frac{n!\,m!}{((m/2)!\,(n/2)!)^2} \frac{1}{\cosh r} (\frac{\tanh r}{2})^{(m+n)} \times \\ \qquad ({}_2F_1(-\frac{n}{2}, -\frac{m}{2}; \frac{1}{2}; -\frac{1}{(\sinh r)^2}))^2 & \text{for m \& n even} \\ \frac{n!m!}{(((m-1)/2)!((n-1)/2)!)^2} \frac{1}{(\cosh r)^3} (\frac{\tanh r}{2})^{(m+n-2)} \times \\ \qquad ({}_2F_1(-\frac{n-1}{2}, -\frac{m-1}{2}; \frac{3}{2}; -\frac{1}{(\sinh r)^2}))^2 & \text{for m \& n odd} \\ 0 & \text{otherwise} \end{cases}$$

is the population distribution of the squeezed number state and ${}_2F_1$ is the Gaussian hypergeometric function.

The squeezed thermal state is prepared by applying the squeezing operation to a thermal state. We validate the preparation procedure by fitting the temporal evolution of the internal state (inset of Supplementary Figure 3**b**) to Supplementary Equation (5), while allowing the fit parameters $p(n)$ for n ranging from 0 to 13 to vary, constrained only by $\sum_n p(n) = 1$. The extracted population distribution is compared to the one predicted by Supplementary Equation 10 with the expected

values of r and $\bar{n}$. The example of such measurement is shown in Supplementary Figure 3 **b** for squeezed thermal state with $r = 1.2$ and $\bar{n} = 0.77$.

Calibration of experimental initial conditions. In Supplementary Figure 4 we show the results of the calibration of the thermal mean phonon numbers and squeezing parameter.

The calibration presented in Supplementary Figure 4(**a-c**) was used to obtain the initial mean phonon numbers shown in main text Fig.2(**a,b,d**) and Fig. 3(**a-f**) [1]. For the experiments performed with lower trap frequencies, for which results are shown in main text Fig. 3(**h-k**) and Fig. 4 the initial mean phonon numbers of the cold mode were obtained directly from the measured $p_\uparrow$ at $\tau = 0$ with the help of Eq. (7) from Methods. For the data taken with squeezed work mode in main text Fig. 3(**h-k**), the initial values for the hot mode were measured once using same number of steps at the beginning of every evolution branch. The work mode initial thermal mean phonon number was measured independently and the values of r were obtained from the calibration shown at Supplementary Figure 4**d**. For the data presented in main text Fig. 4 the work mode initial mean phonon numbers were measured as described above for every individual point while the hot mode phonon number was measured after all the points were taken following the same procedure.

[1]Data presented at main text Fig. 2**c** was taken on different date and used different calibration, which was obtained in exactly the same way. The calibration equations for this data are: $\bar{n}_\mathrm{h} = 0.025 + 0.075(5) \cdot \mathrm{step}$ for the hot mode, $\bar{n}_\mathrm{w} = 0.035 + 0.178(8) \cdot \mathrm{step}$ for the work mode and $\bar{n}_\mathrm{c}^L = 0.044 + 0.075(5) \cdot \mathrm{step}$ $\left(\bar{n}_\mathrm{c}^H = 0.044 + 0.172(8) \cdot \mathrm{step}\right)$ for the cold mode with low (high) power in the lattice.

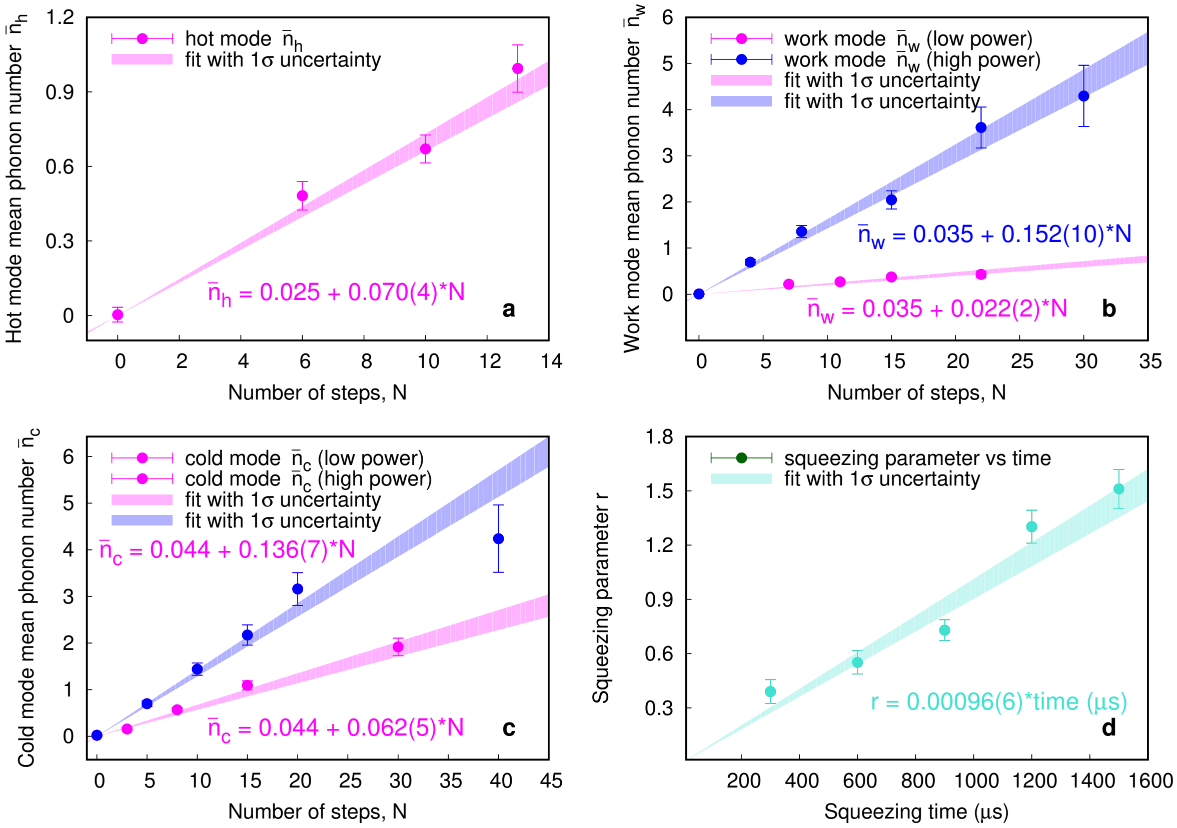

Supplementary Figure 4: **Calibration of the thermal and squeezed state parameters for all modes.** **a.** Dependence of $\bar{n}_{\rm h}$ in the hot mode on the number of steps in the preparation sequence. **b.** Same for work mode $\bar{n}_{\rm w}$ for two different lattice depths. The different depths were employed to cover wider range of initial temperatures. **c.** Same for cold mode. The offset values in the fit functions represent independently measured residual mean phonon numbers after sideband cooling. **d.** Squeezing parameter r of the work mode versus application time of the optical lattice running at $2\omega_{\rm w}$ and trap being set to lower frequency (see Methods). The values of r were extracted by fitting squeezed thermal state evolution with Supplementary Equation 10 with both $\bar{n}$ and r being fit parameters. The error bars represent the standard error of the fit of Supplementary Equation 5 with either thermal (panels **a-c**) or squeezed thermal (panel **d**) to the internal state evolution.

Supplementary Note 4. Error analysis

At each experimental run we probe only one mode of interest during motional state detection. It is the “hot” mode for the data at main text Fig. 2, and “cold” mode for the rest of the data in the main manuscript. Each experimental run provides us with the probability $p_{\uparrow}^{(exp)}$ to find the ion in state $|\uparrow\rangle$ after the red sideband pulse, along with its statistical uncertainty $\delta p_{\uparrow}^{(exp)} = p_{\uparrow}^{(exp)}(1 - p_{\uparrow}^{(exp)})/\sqrt{N}$. This uncertainty propagates to the final value of $n_{exp}(\tau)$ via the function $f(p_{\uparrow}^{(exp)})$ given by Eq. (8) (see Methods), resulting in statistical error $\delta n^{(exp)} = 0.5((f(p_{\uparrow}^{(exp)} + \delta p_{\uparrow}^{(exp)}) - (f(p_{\uparrow}^{(exp)} - \delta p_{\uparrow}^{(exp)}))$. This error is determined for every evolution time τ and is combined with the instrumental error (see below), and is plotted in Fig. 3(**a-e,h-k**) of the main text.

To propagate this error for the data presented in main text Fig. 3(**g,l**), we have to determine the error of the mean phonon number in the asymptotic steady state. We approximate the asymptotic steady state value with the average of the last n points for $\tau > 240\ \mu s$ of the time evolution shown in main text Fig. 3(**a-e**) or $\tau > 600\ \mu s$ for the evolution branches shown in main text Fig. 3(**h-k**). Its error is then given as the error of the mean $\delta_{ss} = \sigma/\sqrt{n}$, where σ is the variance of the points. Together with the error of the initial mean phonon number δ_{in} evaluated at $\tau = 0$, the final statistical error for the difference of initial and steady state values plotted in main text Fig. 3(**g,l**) is given by $\sqrt{\delta_{in}^2 + \delta_{ss}^2}$.

For the data presented in main text Fig. 2(**a-d**) the horizontal error bar is determined by the error of the fit to the calibration data of the ”cold” mode (see Supplementary Figure 4). As before, the vertical error bar is determined by $\sqrt{\delta_{in}^2 + \delta_{ss}^2}$.

As mentioned in the main text, the data in Fig. 2**e** is obtained by fitting data shown on the main text Fig. 2**(a-d)** with $\epsilon_{\rm h}$ derived from numerical simulations of the Hamiltonian given by Eq. (1) of the main text. To obtain the fitting function we calculate $\epsilon_{\rm h}$ as the function of initial mean phonon numbers for about 61000 different values of ($\bar{n}_{\rm h}^{\rm (in)}$, $\bar{n}_{\rm w}^{\rm (in)}$, $\bar{n}_{\rm c}^{\rm (in)}$) and interpolate the resulting $\epsilon_{\rm h}(\bar{n}_{\rm h}^{\rm (in)}, \bar{n}_{\rm w}^{\rm (in)}, \bar{n}_{\rm c}^{\rm (in)})$ with third order polynomials. We use this interpolation function to find the best fit for the data points in main text Fig. 2**(a-d)** in terms of $n_{\rm h}^{\rm (in)}$ and $n_{\rm w}^{\rm (in)}$ and determine $n_{\rm c}^{(eq)}$ for which $\epsilon_{\rm h}(n_{\rm c}^{\rm (in)}) = 0$. The resulting uncertainty of $n_{\rm c}^{(eq)}$ is calculated by propagating the errors of the fit parameters $n_{\rm h}^{\rm (in)}$ and $n_{\rm w}^{\rm (in)}$ using covariance matrix obtained by the fit procedure. The horizontal error bar is given by the uncertainty of the calibration of the "work" mode mean phonon number.

The measurement presented in main text Fig. 4 is taken with a different experimental sequence. As we are only interested in the difference between the initial point and the value at evolution minimum, we alternate the evolution time between $\tau = 0$ and τ_{min} within one experimental run. The horizontal error bar is again given by the uncertainty of work mode calibration, and the vertical error is propagated to the mean phonon number difference from statistical uncertainties of $p_{\uparrow}^{(exp)}(\tau = 0)$ and $p_{\uparrow}^{(exp)}(\tau_{min})$.

Finally, we discuss the effects of "spectator" modes on the preparation and measurement procedures. When we work with the radial modes of motion, all the operations with the target "cold" mode can be affected by the presence of adjacent "work" mode (see Supplementary Figure 1). To characterize this influence during the preparation procedure, we prepare both modes in the vacuum

state, followed by populating the "work" mode with some phonon number and measuring the induced phonon number in the "cold" mode. The results are shown in Supplementary Figure 5. We see that without the trilinear interaction, the population of the cold mode remains in the vacuum state even for highly populated "work mode". We are thus confident that the prepared initial mean phonon numbers in radial modes are not affected by the presence of spectators.

During motional state detection, the contribution of the spectator mode changes the detected ion brightness at the "cold" mode frequency. This change would remain constant if the mean phonon number in the spectator mode remains the same. However, during the interaction, the mean phonon number in the spectator mode may change. The overall change in detected ion brightness thus depends on the difference of the mean phonon number of the spectator mode, in particular the "work" mode, before and after the interaction. Although the difference of the mean phonon number in the "work" mode is not directly available, we note that due to the symmetry of the interaction Hamiltonian, it should be the same as the measured changes in mean phonon number of the "cold" mode. We compute the changes in "work" mode numbers and corresponding brightness change from measured $\bar{n}_{\mathrm{c}}^{(\mathrm{in})}$ at every $\bar{n}_{\mathrm{c}}(\tau)$ (see Supplementary Figure 6 as an illustration) and propagate this systematic error to the final error of the detected mean phonon numbers. This is done by converting the estimated brightness change into the phonon number with the help of Eq. (8) in Methods and adding it to the statistical error in the final $\bar{n}_{\mathrm{c}}(\tau)$. We outline the contributions from this effect to the measured long time average values in the Supplementary Table 3 below. The probing time for all the data here is $t_{rsb} = \pi/(3\Omega) \approx 50\,\mu s$. We can conclude that the contributed change in the detected mean phonon number difference of the "cold" mode is negligible and the

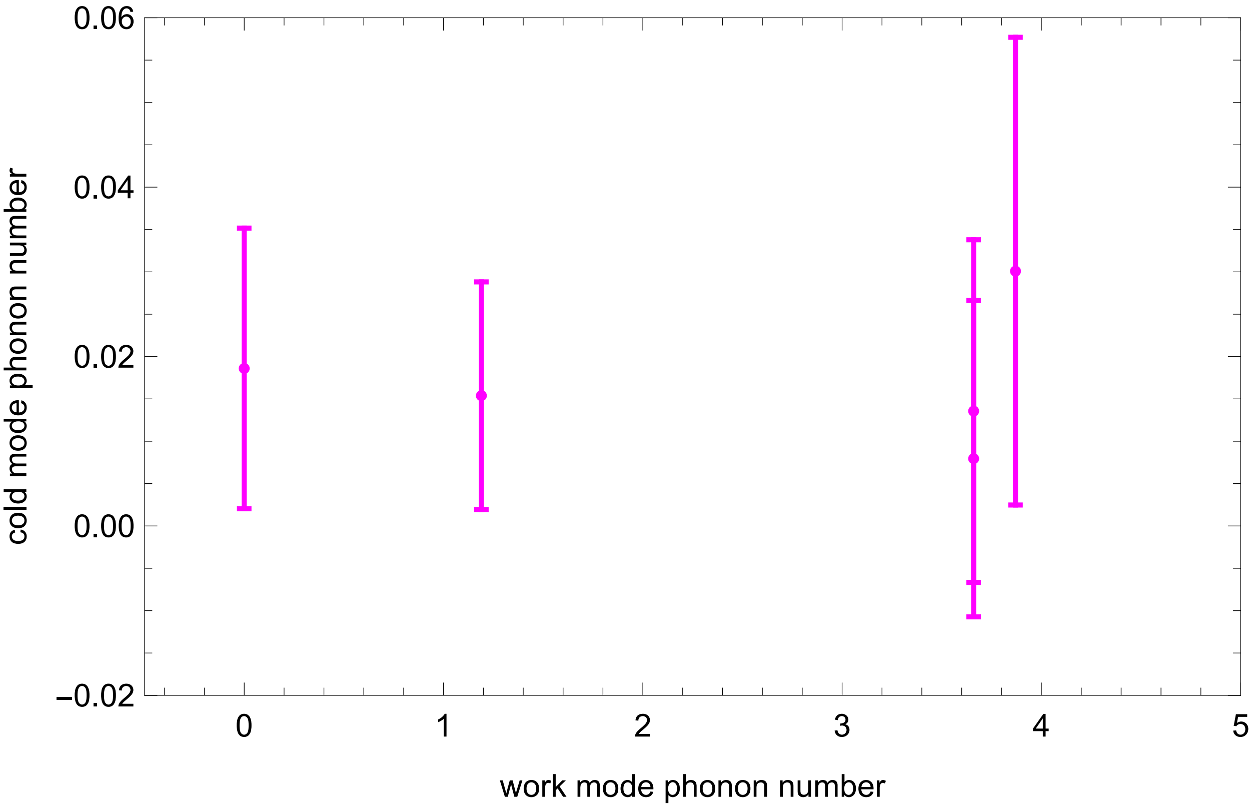

Supplementary Figure 5: Influence of “work” mode population on the “cold” mode without tri-linear interaction. The vacuum state of the “cold” mode does not change with the increase of the energy in the spectator “work” mode. The error bars are the standard error of the fit of Supplementary Equation 5 used to extract the mean phonon number.

results shown in main text Fig. 3 **(h,l)** and Fig. 4 are not affected by this effect.

Supplementary Note 5. Steady state values

To compensate for slow systematic drifts of the initial mean phonon numbers we plot the measured mean phonon numbers of the refrigerator modes relative to the steady state values. This approach does not affect any of our conclusions, but provides better visualization of data. In the Supplementary Tables 4, 5 we show the measured steady state values as well as the initial conditions for all

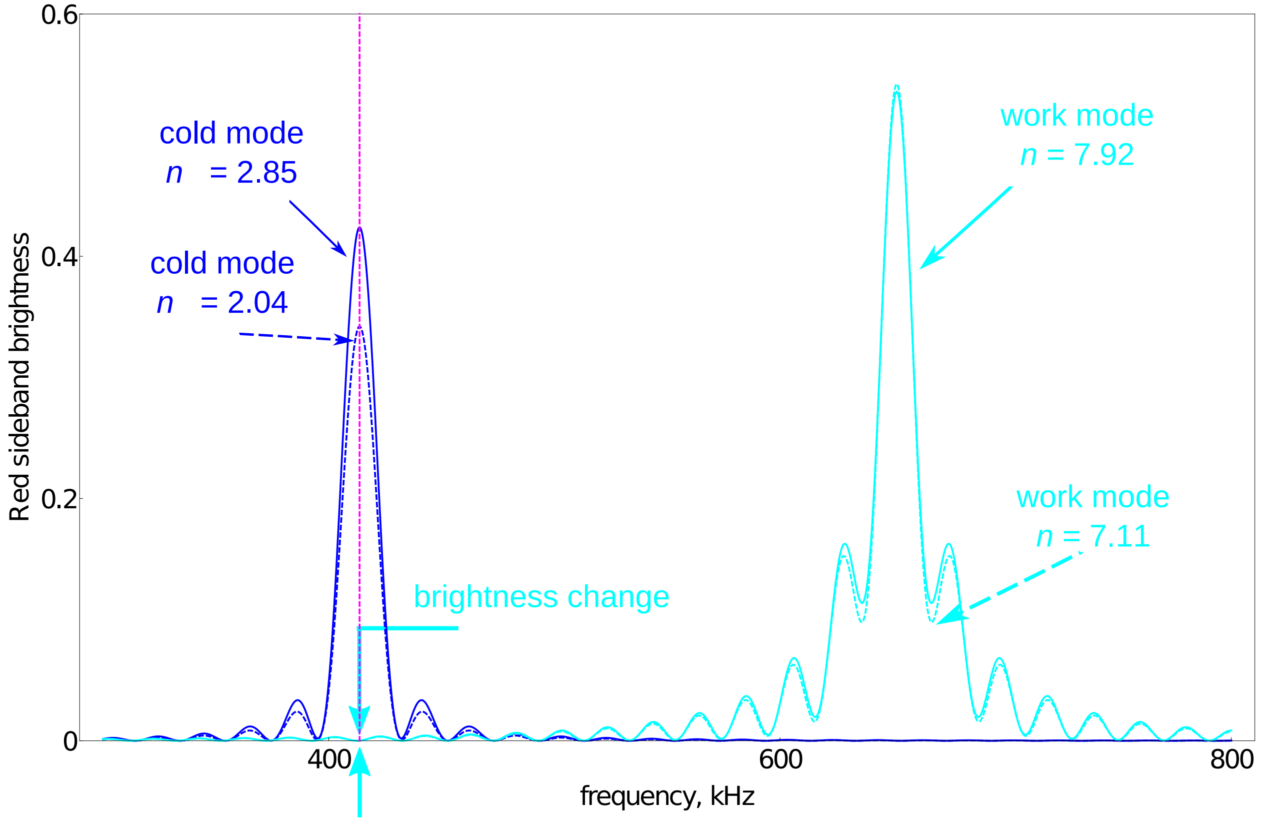

Supplementary Figure 6: Calculated spectator ("work") mode contributions to the signal detected at target ("cold") mode. The depicted situation corresponds to the point with largest initial work number, shown in main text Fig. 4. The detected "cold " mode $\bar{n}_{\mathrm{c}}$ change of ≈ 0.8 defines the change in the "work" mode energy, and its brightness contribution to the detected signal. For this data, the contribution would result in negligible error in the final "cold" mode mean phonon number.

Off-resonant mode frequency difference	mean phonon number			Average spectator brightness change in steady state	Corresponding instrumental error
MHz	initial		difference to steady state	$\Delta p_{\uparrow}^{spec}$	Δn_{c}^{spec}
	cold	work	cold		
Figure 3 (a-g)					
0.316	2.93	0.19	-0.0016	$8.3 \cdot 10^{-8}$	$1.86 \cdot 10^{-6}$
	2.49	0.37	-0.219	$1.94 \cdot 10^{-5}$	$2.50 \cdot 10^{-4}$
	2.40	0.67	-0.218	$1.86 \cdot 10^{-5}$	$2.36 \cdot 10^{-4}$
	2.55	1.10	0.015	$-1.28 \cdot 10^{-6}$	$-1.59 \cdot 10^{-5}$
	2.66	2.16	0.079	$-6.88 \cdot 10^{-6}$	$-8.42 \cdot 10^{-5}$
	2.34	4.44	0.235	$-1.48 \cdot 10^{-5}$	$-1.71 \cdot 10^{-4}$
Figure 3 (h-l)					
0.238	3.01	0.5	-0.255	$8.31 \cdot 10^{-6}$	$1.22 \cdot 10^{-4}$
	2.81	1.94	0.057	$-1.08 \cdot 10^{-6}$	$-1.41 \cdot 10^{-5}$
	2.72	4.54	0.27	$-1.86 \cdot 10^{-6}$	$-2.14 \cdot 10^{-5}$
	2.60	6.83	0.347	$-9.26 \cdot 10^{-7}$	$-1.11 \cdot 10^{-5}$
Figure 4					
0.238	3.14	1.3	-0.0435	$1.23 \cdot 10^{-6}$	$1.83 \cdot 10^{-5}$
	2.88	1.8	0.420	$-9.85 \cdot 10^{-6}$	$-1.3 \cdot 10^{-4}$
	2.73	4.2	0.650	$-7.27 \cdot 10^{-6}$	$-7.91 \cdot 10^{-5}$
	2.70	4.9	0.684	$-5.98 \cdot 10^{-6}$	$-6.57 \cdot 10^{-5}$
	2.85	7.9	0.828	$-2.05 \cdot 10^{-6}$	$-1.95 \cdot 10^{-5}$

Supplementary Table 3: "Spectator" (work) mode contributions (right column) to the ion brightness

the relevant experiments. The steady state values $\bar{n}^{(\mathrm{ss})}$ that were used to generate main text Fig. 2, 3**(a-f)**, were obtained by averaging the measured $\bar{n}(\tau)$ for $\tau > 240\,\mu$s. For main text Fig. 4 the averaging was performed for $\tau > 600\,\mu$s.

Figure 2 (a)		
Number of steps (h, w, c)	$\bar{n}_i^{in}(h, w, c)$	$\bar{n}_{\mathrm{h}}^{ss}$
$(9, 29\mathrm{H}, 7\mathrm{L})$	$(0.66(4), 4.44(29), 0.48(4))$	$0.47(1)$
$(9, 29\mathrm{H}, 14\mathrm{L})$	$(0.66(4), 4.44(29), 0.91(7))$	$0.61(2)$
$(9, 29\mathrm{H}, 10\mathrm{H})$	$(0.66(4), 4.44(29), 1.40(7))$	$0.72(1)$
$(9, 29\mathrm{H}, 13\mathrm{H})$	$(0.66(4), 4.44(29), 1.81(9))$	$0.82(2)$
$(9, 29\mathrm{H}, 17\mathrm{H})$	$(0.66(4), 4.44(29), 2.36(12))$	$0.91(4)$
$(9, 29\mathrm{H}, 20\mathrm{H})$	$(0.66(4), 4.44(29), 2.76(14))$	$1.07(5)$

Figure 2 (b)		
Number of steps (h, w, c)	$\bar{n}_i^{in}(h, w, c)$	$\bar{n}_{\mathrm{h}}^{ss}$
$(9, 16\mathrm{H}, 7\mathrm{L})$	$(0.66(4), 2.47(0.16), 0.48(4))$	$0.50(2)$
$(9, 16\mathrm{H}, 14\mathrm{L})$	$(0.66(4), 2.47(0.16), 0.91(7))$	$0.63(1)$
$(9, 16\mathrm{H}, 10\mathrm{H})$	$(0.66(4), 2.47(0.16), 1.40(7))$	$0.65(2)$
$(9, 16\mathrm{H}, 13\mathrm{H})$	$(0.66(4), 2.47(0.16), 1.81(9))$	$0.73(3)$
$(9, 16\mathrm{H}, 17\mathrm{H})$	$(0.66(4), 2.47(0.16), 2.36(12))$	$0.73(3)$
$(9, 16\mathrm{H}, 20\mathrm{H})$	$(0.66(4), 2.47(0.16), 2.76(14))$	$0.75(3)$

Figure 2 (c)		
Number of steps (h, w, c)	$\bar{n}_i^{in}(h, w, c)$	$\bar{n}_{\mathrm{h}}^{ss}$
$(8, 10, 8\mathrm{L})$	$(0.63(4), 1.82(8), 0.64(4))$	$0.44(2)$
$(8, 10, 15\mathrm{L})$	$(0.63(4), 1.82(8), 1.17(8))$	$0.54(1)$
$(8, 10, 23\mathrm{L})$	$(0.63(4), 1.82(8), 1.77(12))$	$0.58(1)$
$(8, 10, 29\mathrm{L})$	$(0.63(4), 1.82(8), 2.22(15))$	$0.66(3)$
$(8, 10, 35\mathrm{L})$	$(0.63(4), 1.82(8), 2.67(18))$	$0.69(2)$
Figure 2 (d)		
Number of steps (h, w, c)	$\bar{n}_i^{in}(h, w, c)$	$\bar{n}_{\mathrm{h}}^{ss}$
$(9, 7\mathrm{H}, 7\mathrm{L})$	$(0.66(4), 1.10(7), 0.48(4))$	$0.49(1)$
$(9, 7\mathrm{H}, 14\mathrm{L})$	$(0.66(4), 1.10(7), 0.91(7))$	$0.51(1)$
$(9, 7\mathrm{H}, 10\mathrm{H})$	$(0.66(4), 1.10(7), 1.40(7))$	$0.60(2)$
$(9, 7\mathrm{H}, 13\mathrm{H})$	$(0.66(4), 1.10(7), 1.81(9))$	$0.65(2)$
$(9, 7\mathrm{H}, 17\mathrm{H})$	$(0.66(4), 1.10(7), 2.36(12))$	$0.62(1)$
$(9, 7\mathrm{H}, 20\mathrm{H})$	$(0.66(4), 1.10(7), 2.76(14))$	$0.74(1)$

Supplementary Table 4: Measured steady state values for the presented data. The left column shows the number of phase modulating steps for thermal state preparation in each mode. “H” or “L” letters indicate high or low power of the optical lattice. Initial mean phonon numbers obtained from calibration measurements are shown in the middle column. The measured steady state values are presented in the right column.

Figure 3 (a-f)		
Number of steps (h, w, c)	$\bar{n}_i^{in}(h, w, c)$	$\bar{n}_{\mathrm{c}}^{ss}$
$(9, 29\mathrm{H}, 19\mathrm{H})$	$(0.66(4), 4.44(29), 2.63(13))$	$2.17(3)$
$(9, 14\mathrm{H}, 19\mathrm{H})$	$(0.66(4), 2.16(14), 2.63(13))$	$2.63(3)$
$(9, 7\mathrm{H}, 19\mathrm{H})$	$(0.66(4), 1.10(7), 2.63(13))$	$2.59(3)$
$(9, 29\mathrm{L}, 19\mathrm{H})$	$(0.66(4), 0.67(6), 2.63(13))$	$2.67(4)$
$(9, 15\mathrm{L}, 19\mathrm{H})$	$(0.66(4), 0.37(3), 2.63(13))$	$2.77(5)$
$(9, 7\mathrm{L}, 19\mathrm{H})$	$(0.66(4), 0.19(1), 2.63(13))$	$2.99(8)$
Figure 3 (h-k)		
Number of steps $(h, w, \mathbf{squeezing\,time}, c)$	$\bar{n}_i^{in}(h, w, \mathbf{r}, c)$	$\bar{n}_{\mathrm{c}}^{ss}$
$(9, 8, \mathbf{1400}\,\mu s, 19\mathrm{H})$	$(0.47(6), 0.50(5), \mathbf{1.34(8)}, 2.60(3))$	$2.29(6)$
$(9, 8, \mathbf{1200}\,\mu s, 19\mathrm{H})$	$(0.52(6), 0.50(5), \mathbf{1.15(7)}, 2.72(4))$	$2.49(4)$
$(9, 8, \mathbf{800}\,\mu s, 19\mathrm{H})$	$(0.52(6), 0.50(5), \mathbf{0.77(4)}, 2.81(4))$	$2.80(3)$
$(9, 8, \mathbf{0}\,\mu s, 19\mathrm{H})$	$(0.46(6), 0.50(5), \mathbf{0}, 3.01(4))$	$3.31(6)$

Supplementary Table 5: Measured steady state values for the presented data. The left column shows the number of phase modulating steps for thermal state preparation in each mode and the squeezing time used to prepare a squeezed thermal state. Initial mean phonon numbers obtained either from calibration or direct measurements are shown in the middle column. The measured steady state values are presented in the right column.

Supplementary References

1. Marquet, C., Schmidt-Kaler, F. & James, D. Phonon–phonon interactions due to non-linear effects in a linear ion trap. *Applied Physics B* **76**, 199–208 (2003).

2. Lee, A. C. *et al.* Engineering large stark shifts for control of individual clock state qubits. *Phys. Rev. A* **94**, 042308 (2016).

3. Meekhof, D. M., Monroe, C., King, B. E., Itano, W. M. & Wineland, D. J. Generation of nonclassical motional states of a trapped atom. *Phys. Rev. Lett.* **76**, 1796–1799 (1996).

4. Loudon, R. *The Quantum Theory of Light* (Oxford University Press, Oxford, U.K., 2000), 3 edn.

5. Leibfried, D., Blatt, R., Monroe, C. & Wineland, D. Quantum dynamics of single trapped ions. *Rev. Mod. Phys.* **75**, 281–324 (2003).

6. Kim, M., De Oliveira, F. & Knight, P. Properties of squeezed number states and squeezed thermal states. *Physical Review A* **40**, 2494 (1989).

7. Marian, P. Higher-order squeezing and photon statistics for squeezed thermal states. *Physical Review A* **45**, 2044 (1992).
